# Therapeutic Potential of Umbilical Cord MSC-Derived Exosomes in a Severe Dry Eye Rat Model: Enhancing Corneal Protection and Modulating Inflammation

**DOI:** 10.3390/biomedicines13051174

**Published:** 2025-05-11

**Authors:** Sze-Min Chan, Chris Tsai, Tai-Ping Lee, Zih-Rou Huang, Wei-Hsiang Huang, Chung-Tien Lin

**Affiliations:** 1 Graduate Institute of Veterinary Medicine, School of Veterinary Medicine, National Taiwan University, Taipei 106319, Taiwan; d06629002@ntu.edu.tw; 2 Institute of Veterinary Clinical Sciences, School of Veterinary Medicine, National Taiwan University, Taipei 106319, Taiwan; 3 BIONET Therapeutics Corp., Taipei 114065, Taiwan; 4 Graduate Institute of Molecular and Comparative Pathobiology, School of Veterinary Medicine, National Taiwan University, Taipei 106319, Taiwan; 5 Department of Ophthalmology, National Taiwan University Veterinary Hospital, College of Bioresources and Agriculture, National Taiwan University, Taipei 106319, Taiwan

**Keywords:** umbilical cord MSC-derived exosomes, dry eye disease, rat model, corneal epithelium

## Abstract

**Background/Objectives**: Dry eye disease (DED) is a multifactorial inflammatory disease that disrupts the ocular surface, causing tear film instability, epithelial damage, and chronic inflammation. Mesenchymal stem cell-derived exosomes (MSC-exos) are promising therapeutics with immunomodulatory and regenerative properties. This study investigates the therapeutic effects of umbilical cord MSC-derived exosomes (UCMSC-exos) in a severe dry eye model, induced by a surgical resection of the infra-orbital (ILG) and extra-orbital lacrimal gland (ELG) in rats. **Methods**: Clinical evaluations, including tear volume measurement, slit lamp biomicroscopy, fluorescein staining, and spectral domain optical coherence tomography (SD-OCT), were performed to assess corneal neovascularization, corneal abrasion, and epithelial/stromal thickness. Histopathological analysis, immunohistochemistry, and mRNA gene expression were conducted to evaluate corneal tissue changes and inflammatory marker expression. **Results**: The results show that the treatment group exhibited significantly reduced corneal neovascularization compared to the control group (*p* = 0.030). During the first month, the Exo group also had a significantly lower corneal fluorescein staining area (*p* = 0.032), suggesting accelerated wound healing. SD-OCT analysis revealed that the corneal epithelial thickness in the treatment group was closer to normal levels compared to the control group (*p* = 0.02 and *p* = 0.006, respectively). UCMSC-exos treatment also modulated the expression of α-SMA and apoptosis in the cornea. Additionally, the gene expression of inflammatory cytokines (IL-1β and TNF-α) were downregulated. **Conclusions**: These findings suggest that MSC-exosome therapy offers a novel, cell-free regenerative approach for managing severe DED, modulating inflammatory response.

## 1. Introduction

### 1.1. Dry Eye Disease (DED)

Dry eye disease (DED), also known as Keratoconjunctivitis Sicca, is a complex, multifaceted inflammatory disease of the ocular surface that involves intrinsic and extrinsic factors promoting the development of the disease [1]. Intrinsic factors of the host, such as autoimmune conditions, female androgenic effect, and aging, are attributed to glandular secretory dysfunction, neural sensitization, tear instability, and later corneal surface desiccation. Extrinsic factors include environmental stress, usage of preservative ocular products, and surgical intervention, such as LASIK surgery [1,2]. In general, DED is divided into decreased tear secretion (aqueous-deficient DED) and increased tear evaporation (hyper-evaporative DED) [1,3,4]. During the development of the disease, an inflammatory cycle is initiated when there are tear composition, osmolarity, and volume changes (either qualitatively or quantitatively), causing superficial keratitis and keratoconjunctivitis [3,5]. This triggers the production of innate inflammatory mediators (including interleukin IL-1β, TNF-α, and IL-6) by ocular-surface epithelial cells and antigen-presenting cells (APCs) on the cornea’s surface, and conjunctiva (macrophages, monocytes, and dendritic cells) that stimulate the production of matrix metalloprotease (MMP), such as MMP-3 and MMP-9, which causes the apoptosis of the epithelial cells [6,7,8,9]. Simultaneously, there is an upregulation of natural killer (NK) cells and neutrophils that contribute to more inflammatory response and phagocytoses [10]. Damaged/apoptotic cells further release interleukins that upregulate other inflammatory cells and dendritic cell maturation [4,7,11]. These mediators, combined with exposure to autoantigens, generate an adaptive T-cell-mediated response [8,12,13]. The release of inflammatory cytokines and mediators into tears worsens tear film stability by hyperosmolarity, where they then create a vicious cycle of mechanisms that progressively damage the ocular surface and lacrimal apparatus [1,3].

### 1.2. Umbilical Cord Mesenchymal Stem Cell-Derived Exosome

By definition, exosomes are the smallest subtype (approximately 40–150 nm) of extracellular vesicles that are inward-budding endosomes from most eukaryotic cells [14]. Mesenchymal stem cells (MSCs) are pluripotent stem cells known for their regenerative abilities and their capacity to differentiate into various cell types, such as osteoblasts, chondrocytes, adipocytes, and other cell types [15,16,17]. According to the criteria established by the International Committee, human MSCs are defined and characterized by their adherence to plastic surfaces under standard culture conditions; expression of surface markers CD105, CD73, and CD90; and lack of expression of hematopoietic markers, such as CD45, CD34, CD14, CD11b, CD79a, CD19, and HLA-DR. In vitro, these cells are capable of differentiating into osteogenic, adipogenic, and chondrogenic lineages [18]. Increasing evidence indicates that MSCs primarily exert their immunomodulatory function through the secretion of paracrine factors, notably exosomes [19,20]. MSC-exos have been shown to suppress the activation of pro-inflammatory M1 macrophages and encourage their transition into the anti-inflammatory M2 phenotype. This shift results in a reduction in VEGF-A, IFN-γ, IL-12, and TNF-α levels, while extracellular IL-10 levels increase [21]. Additionally, MSC-exos exert an immunosuppressive influence on natural killer (NK) cells by inhibiting their proliferation, activation, and cytotoxic functions. In human graft-versus-host disease (GVHD) studies, Kordelas and colleagues observed that MSC-exos diminished the production of IFN-γ and TNF-α by activating NK cells. This led to reduced NK cell cytotoxicity and a subsequent decrease in inflammatory responses [22]. It was found that transgene-free human-induced pluripotent stem cell (iPSCs)-derived EVs could suppress the activation of immune cells and expression of proinflammatory factors, hence preventing the progression of Sjögren’s syndrome (SS) by inhibiting the differentiation of follicular helper T (Tfh) and Th17 cells [17,23].

Currently, orthodox treatment with artificial tears and other lubricating eyedrops helps relieve symptoms and facilitate the healing of the ocular surface, though ameliorating the progressive and severe keratoconjunctivitis mechanism remains the crucial objective [24,25]. Current medications that are immunoregulators or inflammatory suppressors, including cyclosporine A, corticosteroids, tacrolimus, tetracycline derivatives, and autologous serum, have been effective for the management of dry eye and lead to measurable clinical improvement [24,26]. Although corticosteroids and immunosuppressants are effective in managing severe DED, long-term use may lead to adverse effects, including a higher risk of infections and impaired ability to repair and regenerate the damaged ocular surface [25,27]. MSCs and their secretome have been recognized as potential therapeutic agents for inflammatory eye diseases, including severe DED, due to their ability to produce a large number of immunomodulatory factors. These factors can effectively regulate harmful immune responses in the eyes and reduce systemic inflammation without causing significant adverse effects [28,29,30].

In this study, we aimed to establish a dry eye rat model via total lacrimal gland resection, mimicking the pathophysiology of hyperosmotic, severe dry eye. This approach allows for investigating ocular-surface changes, immune responses, and potential therapeutic interventions in a controlled experimental setting. The application of topical umbilical cord-derived mesenchymal stem cell (UCMSC)-derived exosomes (UCMSC-exos) in the dry eye rat model allows us to evaluate their potential to reduce ocular-surface damage, and modulate inflammatory responses. Additionally, we analyzed histopathological changes and inflammatory marker expression to assess the therapeutic impact of UCMSC-exos on dry eye disease progression.

## 2. Materials and Methods

### 2.1. UCMSC-exos Eye Drop

UCMSC-exos were generously provided by BIONET Therapeutics Corp (Taipei, Taiwan). The exosomes were provided as a ready-to-use formulation, and their production followed the proprietary protocols of the manufacturer. Detailed information on the isolation and characterization methods was not available due to commercial confidentiality. The UCMSCs were of human origin and were validated through tri-lineage differentiation assays, demonstrating adipogenic, chondrogenic, and osteogenic potential. Their surface marker profile was characterized by the expression of CD29, CD90, and CD105, with the absence of hematopoietic markers, including CD45, CD34, and HLA-DR, consistent with the established MSC criteria. For exosome preparation, the cell culture supernatant was collected and concentrated using tangential flow filtration (TFF). The resulting exosome concentrate was then subjected to nanoparticle tracking analysis (NTA) to assess particle size and concentration, as well as to validation for exosomal surface markers and compositional analysis. The particle size of the UCMSC-exos was approximately 121.9 nm. The concentration of the exosomes was tested as 115 × 10^8^ particles/0.5 mL. The UCMSC-exos were positive for exosomal markers CD9^+^, CD63^+^, and CD81^+^, and carry a range of bioactive cytokines and growth factors, including bFGF, PDGF, PEDF, HGF, and TGF-β1.

### 2.2. Animals and Dry Eye Rat Model

Female Sprague Dawley rats aged 6 to 8 weeks were utilized in this study. The animals were maintained under standard laboratory conditions in ventilated cages, with ambient temperatures regulated between 20 °C and 25 °C and a 12 h light/dark cycle. All experimental procedures were conducted in accordance with the guidelines approved by the Institutional Animal Care and Use Committee (IACUC) of the National Taiwan University (NTU-112-EL-00026 and NTU-113-EL-00088).

**Figure 1:**
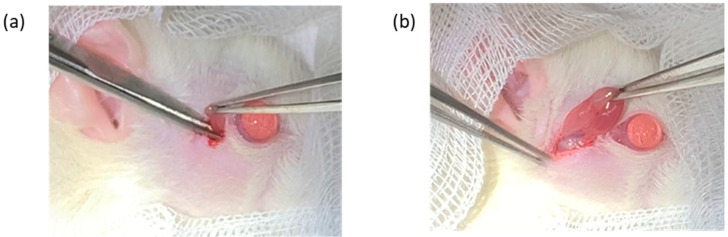
Image of the surgical anatomy of the infraorbital lacrimal gland (**a**) and extra-orbital lacrimal gland (**b**).

The rats were given 2 mg/kg of oral meloxicam (Mobic^®^ Tablets 7.5 mg, Boehringer Ingelheim Ellas A.E., Attica, Greece) and 25 mg/kg of oral amoxicillin (amoxicillin capsules 500 mg, China Chemical & Pharmaceutical Co., Ltd., Taipei, Taiwan) about 1 h prior to the surgery. At the same time, post-operative care with oral medication, including oral meloxicam (1 mg/kg, SID for 2 days, starting from the day after surgery) and amoxicillin (25 mg/kg, BID for 5 days) was administered to all rats. The rats were anesthetized with intraperitoneal 20 mg/kg tiletamine hydrochloride and zolazepam hydrochloride (Zoletil^®^, 500 mg/5 mL, Virbac AH, Inc., Carros, France) in combination with 10 mg/kg Xylazine hydrochloride. Unilateral right infra-orbital and extra-orbital lacrimal gland excisions were performed while the left lacrimal glands were left intact in all groups [31]. In brief, a skin incision was made 2 mm away from the lateral canthus to expose the ILG, which was excised using ophthalmic forceps and tenotomy scissors. The subcutaneous tissue layer was then excised and extended caudally toward the cranioventral part of the ear, exposing the ELG, which was excised. 4-0 Nylon sutures were used to close the surgical incision (Figure 1).

### 2.3. Treatment Regimen and Clinical Evaluation

All the rats underwent ILG + ELG excision surgery. The rats were divided into 2 groups, where the control group did not receive any topical eyedrops and the Exo group received topical UCMSC-exos eyedrops 2 times daily starting at 48 h post-operatively. Approximately 20–30 µL of UCMSC-exos eyedrops were instilled into the right eye each time.

An additional control group (control + PBS) was included to account for any potential lubricating effects of the vehicle solution used in the eyedrops. Rats in this group received 20–30 µL of sterile 1X phosphate-buffered solution (PBS) topically applied to the right eye twice daily. Observations were conducted over a 14-day period. Corresponding data are shown in the Supplementary Materials (Figures S1–S3). Data analysis revealed no statistically significant difference between the control group and the control + PBS group.

A clinical evaluation was performed prior to surgery as a baseline parameter. The evaluations included: tear volume measurement (Meta Biomed Absorbent Paper Points, number 30, Seoul, Republic of Korea), slit lamp biomicroscopy (KOWA SL-17, Torrance, CA, USA), Corneal 1% Fluorescein dye staining, and Spectral Domain Optical Coherence Tomography (SD-OCT) (Spectralis^®^ Heidelberg, Germany). A clinical assessment was performed on the days of surgery (day -2), 0, 7, 14, 21, 28, 35, 42, 49, and 56 after the lacrimal gland excision. The purpose of measuring the tear volume was to ensure a persistent lack of aqueous tear production after the surgery and throughout this study. Slit lamp biomicroscopy was employed to assess morphological changes and detect any pathological alterations in the ocular surface and within the anterior uveal structures. To evaluate corneal barrier integrity, fluorescein staining was conducted. Concurrently, clinical images were captured and subsequently analyzed using image processing software (ImageJ version 1.38x; National Institutes of Health, Bethesda, MD, USA). SD-OCT was conducted utilizing the anterior segment imaging protocol to visualize the cornea. Corneal epithelial and stromal thicknesses were quantified through the system’s proprietary analysis software (Heidelberg Eye Explorer Version 6.3; Heidelberg Engineering, MA, USA). For each eye, three measurements were obtained: one at the central cornea (point 1) and two at equidistant medial and lateral locations (points 2 and 3), situated 1000 µm from the center. Final thickness values were determined by calculating the mean across the three anatomical points (refer to Supplementary Figure S4).

### 2.4. Histopathology and Immunohistochemistry

On days 7, 14, 28, and 56, the rats were euthanized for cornea histopathology evaluation. Following enucleation, ocular tissues were immersed in 10% neutral-buffered formalin for 24 h to ensure fixation. Subsequent tissue processing included vertical sectioning, dehydration through graded alcohols, and embedding in paraffin. Sagittal sections of 4 µm thickness, intersecting both the pupil and optic nerve, were stained using hematoxylin and eosin (H&E) and analyzed under a light microscope. Immunohistochemical analysis of the cornea was carried out following previously established protocols [32]. Immunoreactivity was semi-quantitatively assessed by calculating an immunostaining score, defined as the product of the proportion of positively stained cells (PPs) and staining intensity (SI). The PP was graded as follows: 0 (no staining), 1 (<25% cells positive), 2 (25–50%), 3 (51–75%), and 4 (>75%). The SI was scored as: 0 (no signal), 1 (weak), 2 (moderate), and 3 (strong) [33]. The primary antibody used for detecting α-smooth muscle actin (α-SMA) was ab5694 (Abcam, Waltham, MA, USA), and the TUNEL Assay Kit-BrdU-Red (ab66110) was used in this study.

### 2.5. Quantitative Real-Time PCR

Corneal tissue samples were placed into 2 mL Eppendorf tubes containing stainless-steel beads, followed by the addition of 1 mL TRIzol^TM^ Reagent (Invitrogen, Carlsbad, CA, USA). The samples were homogenized using TissueLyser II (Qiagen, Hilden, Germany) at a frequency of 30 Hz for 30 s, which was repeated three times. RNA was then extracted using the Invitrogen TRIzol^TM^ Plus RNA Purification Kit, following the manufacturer’s instructions. RNA quality and concentration were assessed using an EPOCH ELISA reader (BioTek Instruments, Winooski, VT, USA).

After RNA extraction, qPCR was performed using SYBR Green Master Mix on the LightCycler 480 system (Roche, Basel, Switzerland) with software version 1.5.1.62 SP3. GAPDH was used as an internal control, and two technical replicates were prepared for each sample. Relative gene expression was calculated using the 2^−ΔΔCt^ method. The primer sequences used are listed in List S1.

### 2.6. Statistical Analysis

All statistical analyses were performed using IBM SPSS Statistics for Windows, Version 29 (IBM Corporation, Armonk, NY, USA). Data were assessed for normality prior to further analysis. Group comparisons of clinical parameters over time were conducted using a mixed-model repeated measures analysis. The histopathology, immunohistochemistry, and gene expression analysis were tested with the Univariate General Linear Model test.

## 3. Results

### 3.1. Subsection Effects of UCMSC-exos on the Clinical Findings

#### 3.1.1. Corneal Neovascularization

**Figure 2:**
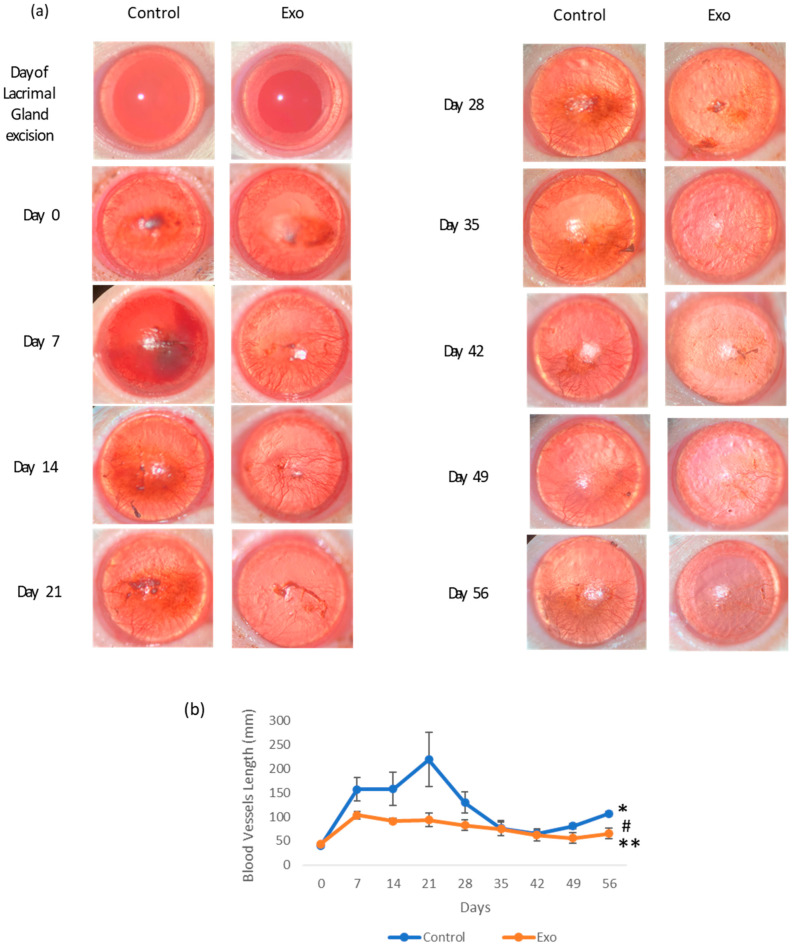
The effects of treatment on corneal neovascularization. (**a**) Photographic images of corneal neovascularization progression of each group from day of lacrimal gland excision (day 2) to day 56. Presence of blood vessels circumferencing the cornea at the perilimbal area at 48 h post-operation (day 0). The Exo group showed a clearer cornea and less dense blood vessels from day 14 to day 56. The control group had a persistent presence of dense blood vessels, even though by day 35, the vessels were more concentrated at the lower equatorial region of the cornea. (**b**) Corneal neovascularization length (mm) changes throughout the 56-day study (* group effects, *p* = 0.03, # days effects, *p* < 0.001, ** group × days interaction effects, *p* = 0.011).

Blood vessels were measured weekly, and photos of the blood vessels’ development on the cornea with the quantification of the total length in a graph illustration are shown in Figure 2. A similar amount of blood vessels was detected on the day DED was established and before treatment started (day 0) in both groups. Blood vessels were seen arising from the perilimbal area with a deposition of small capillaries at the central cornea. The control group showed an initial increase in vessel length, peaking around day 21, followed by a decline. However, by day 49, it showed signs of elevation that persisted till day 56. During the first month of this study, the vessels in the control group densely branched and extended to the equator of the cornea. By the second month, blood vessels were seen to regress to the lower hemisphere of the cornea. The Exo group reached a lower peak on day 7 and gradually declined to a minimum number throughout this study, with the branching evidently reduced even during its peak on days 7 and 14. The linear model revealed the significant main effects of time, F (8, 108.578) = 13.512, *p* < 0.001, suggesting that the total blood vessel length changes significantly across the study period. A significant treatment effect of Exo was observed, F (1, 47.182) = 4.988, *p* = 0.030, indicating that the Exo group exhibited significantly reduced blood vessel formation compared to the control group. Pairwise comparisons revealed a significant mean difference of −41.179 (SE = 18.439, *p* = 0.030). Notably, a trajectory of change was not uniform across groups, as evidenced by a significant days × group interaction (F (8, 108.578) = 2.655, *p* = 0.011).

#### 3.1.2. Corneal Abrasion

**Figure 3:**
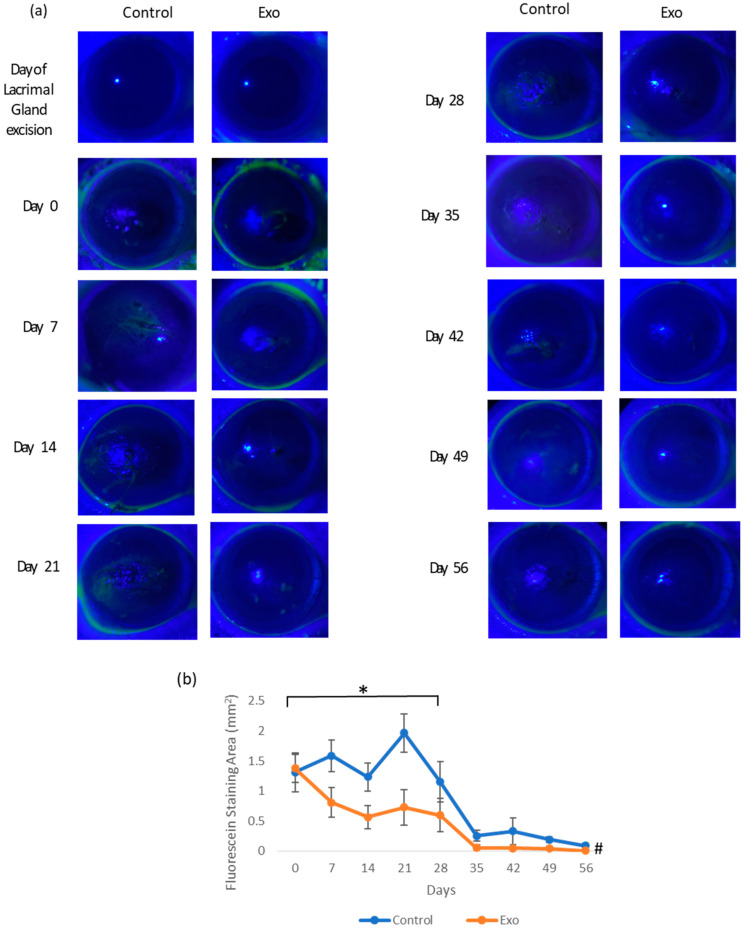
Corneal fluorescein staining. (**a**) Photographic images of corneal fluorescein staining of each group from day of lacrimal gland resection to day 56. The Exo group had minimal staining since day 21 compared to the control group, where the cornea surface was uneven and stained at least till day 35. (**b**) Corneal fluorescein staining area (mm^2^) throughout the 56-day study (* group effects, *p* = 0.032, # days effects, *p* = 0.002).

The intensity of corneal abrasion was determined with corneal fluorescein staining (Figure 3). The corneal fluorescein staining area in the control group was higher during the first month of this study. The Exo group showed a faster decline in fluorescein retention, suggesting accelerated wound healing and mitigated recurrence of abrasion. By day 35, both groups had reduced abrasion areas with the Exo group barely stained. Figure 3a shows the representative images of corneal fluorescein staining throughout this study. The staining pattern demonstrates punctate and multifocal characteristics, predominantly localized in the central region of the cornea. A significant main effects of the time was observed, F (8, 92.742) = 3.290, *p* = 0.002, indicating the fluorescein staining area change significantly over time. The main effects of group and group × days interaction were not significant, indicating that there was no consistent difference in abrasion areas between groups across all time points. However, when we excluded the data from the second month (only testing days 0, 7, 14, 21, and 28), the main effects of the group were significant (F (1, 34.028) = 5.012, *p* = 0.032). This indicated that during the first month, the Exo group had significantly less fluorescein staining areas overall, while still having the same trajectory trends. Pairwise comparison showed a significant mean difference of −0.134 (SE = 0.032, *p* < 0.001).

#### 3.1.3. Corneal Epithelial and Stromal Thickness

**Figure 4:**
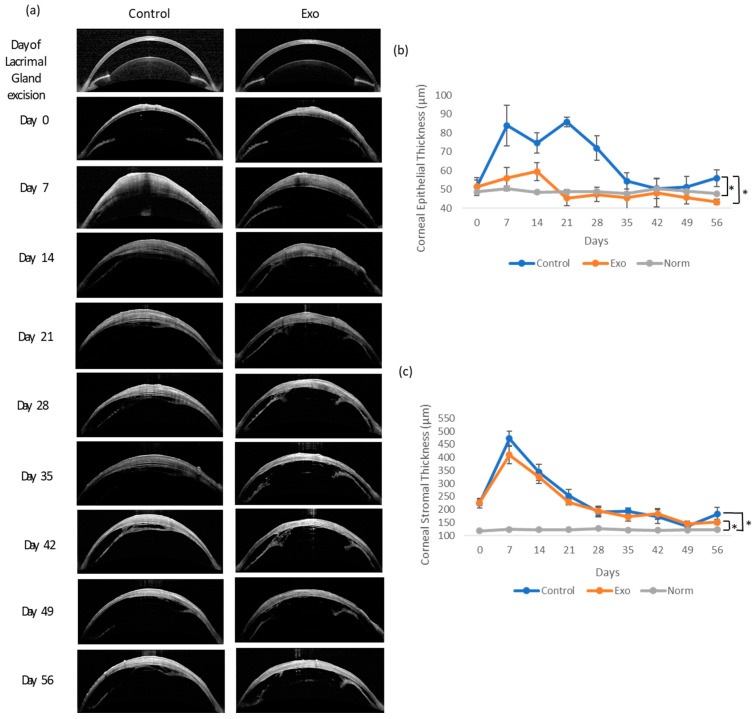
Corneal epithelium and stromal thickness examination and analysis throughout 56 days. (**a**) OCT images of the cornea. Both groups had stromal thickening by day 7 and slowly subsided throughout this study. The control group had focal areas of thickening at the epithelial layer. (**b**) Epithelium thickness (µm) between groups. * Group effects, *p* = 0.003. (**c**) Stromal thickness (µm) between groups. * Group effects, *p* < 0.001.

We compared the epithelial and stromal thickness of both groups to that of normal rats (NORM group) to evaluate the treatment effects on epithelial and stromal cell regulation during DED. Figure 4 shows the OCT images of the cornea and the mean ± SEM of the corneal epithelium and stromal thickness over time. The epithelium of the control group experienced abrupt thickening 7 days after DED establishment, followed by fluctuations at a higher thickness range until day 28. The Exo group demonstrated a more subtle change in thickness over time. The corneal epithelial thickness exhibited significant main effects of the groups (F (2, 46.711) = 6.593, *p* = 0.003). The main effects of time and time x treatment group interaction both tested as not significant, indicating the trajectory of change was similar in both groups over time. Post hoc pairwise comparison revealed that the Norm and Exo groups significantly differed from the control group with a mean difference (MD) = −14.957, SE = 5.242, *p* = 0.02 and MD = −14.923, SE = 4.570, *p* = 0.006, respectively.

The stromal thickness showed a highly significant main effects of the groups, F (1, 41.919) = 14.858, *p* < 0.001. A significant effect of time, F (8, 111.147) = 14.182, *p* < 0.001, indicated that stromal thickness changed significantly across the study period. Importantly, the significant time x group interaction, F (16, 111.805) = 2.830, *p* < 0.001, suggested that the trajectory of change differed between groups. Post hoc pairwise comparison showed both the Exo and control group experienced significant thickening compared to the NORM group (MD = 56.608, SE = 8.506, *p* < 0.001 and MD = 65.808, SE = 8.506, *p* < 0.001, respectively). The thickening of both groups peaked in the first week after DED. In general, the control and Exo groups shared similar trends of thickening throughout this study, except during the first week, where the control group was slightly thicker, and exhibited signs of relapsing in the final week.

### 3.2. Histopathological Findings

#### 3.2.1. Effects of UCMSC-exos on Apoptotic Cells and Neutrophils Infiltration

**Figure 5:**
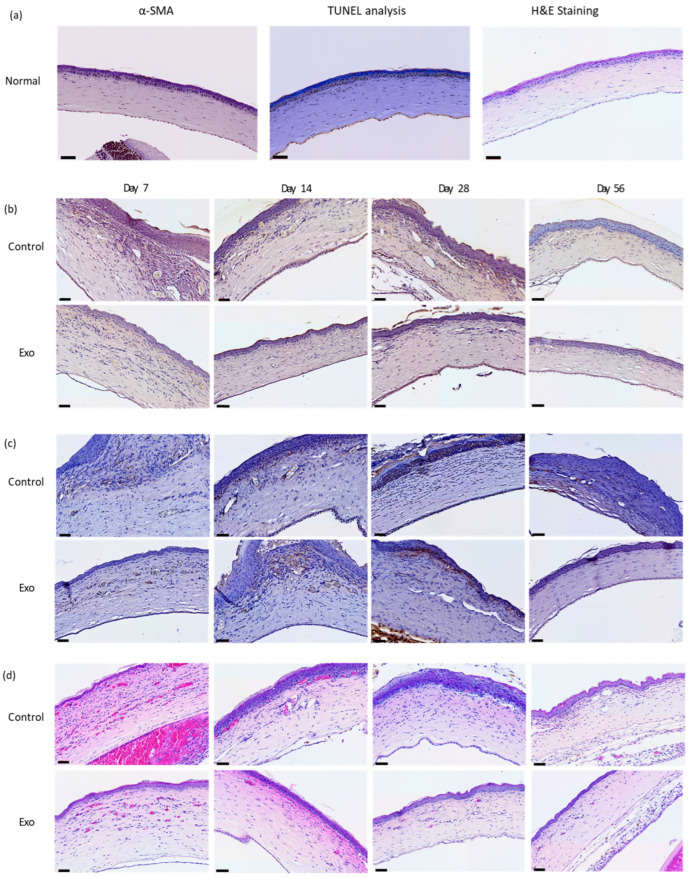
Histopathology and immunohistochemistry staining. (**a**) Normal rats. Generally a lack apoptotic cells, minuscule α-SMA staining at the anterior stroma, and absence of neutrophils in H&E staining. (**b**) TUNEL assay. Apoptotic cells are mainly at the epithelial surface and areas near epithelial basement membrane. (**c**) α-SMA staining. Immnunoreactivity present in the anterior stroma and areas near the epithelial basement membrane. (**d**) H&E-staining neutrophil cell count. Neutrophils infiltrated mainly the anterior half of the stroma, predominantly immediately below the epithelial basement membrane. Scale bar = 50 µm.

**Figure 6:**
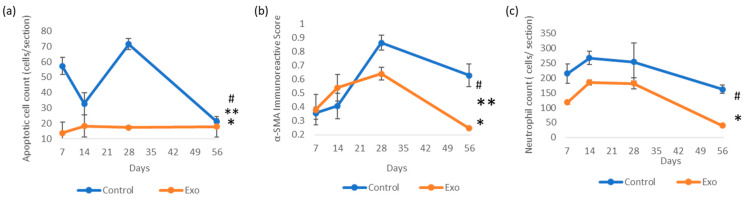
Immunoreactivity scores (IRSs) of (**a**) apoptotic cells (TUNEL assay) (* group effects, *p* < 0.001, # days effects, *p* < 0.001, ** groups × days interaction, *p* < 0.001); (**b**) α-SMA immunohistochemistry staining (* groups effects, *p* = 0.04, # days effects, *p* < 0.001, ** days × groups interaction effects, *p* = 0.008); and (**c**) neutrophil count with H&E staining analysis (* group effects, *p* < 0.001, # days effects, *p* < 0.001).

The control group showed a much higher apoptotic cell count from the beginning of this study (Figure 5). It exhibited trends of decline but peaked again at day 28. The univariate GLM test showed a significant difference in the apoptotic cell count between the control and Exo groups, F (1, 24) = 53.909, *p* < 0.001, η^2^_p_ = 0.692. The main effects of days were also significant, F (3, 24) = 7.854, *p* < 0.001, η^2^_p_ = 0.495, indicating the apoptotic cell count changed significantly across time points. The treatment × days interaction was significant, F (3, 24) = 9.273, *p* < 0.001, η^2^_p_ = 0.537, suggesting that both groups exhibited different trends over time (Figure 6). In the Exo group, the apoptotic cell count remained lower across all time points. The pairwise comparison demonstrated that the Exo group had a significant mean difference of −28.875, (SE = 3.933, *p* < 0.001) than the control group.

We observed a high infiltration of neutrophils, which persisted over time in the control group (Figure 5). It peaked at day 14, followed by a gradual decline. The Exo group had a lower initial neutrophil count, with a sharp decline after day 28. The univariate GLM revealed a significant main effects of group, F (1, 24) = 21.989, *p* < 0.001, η^2^_p_ = 0.478, indicating the Exo group had less neutrophil infiltration. The main effects of time tested significant, F (3, 24) = 8.377, *p* < 0.001, η^2^_p_ = 0.512, indicating neutrophil infiltration changes significantly across time. The insignificant treatment × days interaction suggested that both the groups presented similar trends over time. The Exo group presented significantly fewer neutrophils infiltrating the cornea, with a pairwise comparison showing a highly significant mean difference of −93.563 (SE = 19.162, *p* < 0.001).

#### 3.2.2. Effects of UCMSC-exos on Myofibroblast Regulation

The control group had increasing α-SMA IRS over time, peaking at day 28 (Figure 5 and Figure 6). In comparison, the Exo group had a gradual increment till day 28. Both groups showed declining trends after day 28, though the control group had a higher IRSs. The control group had significantly higher scores compared to the Exo group (F (1, 24) = 4.729, *p* = 0.04, η^2^_p_ = 0.165). The main effects of time were significant as well, F (3, 24) = 10.471, *p* < 0.001, η^2^_p_ = 0.567, indicating the amount of myofibroblasts changed significantly across time points. The group x days interaction was also significant, F (3, 24) = 5.032, *p* = 0.008, η^2^_p_ = 0.386, suggesting both groups had different trends over time. Pairwise comparison between groups also demonstrated that the Exo group had a significant mean difference of −0.113 (SE = 0.052, *p* = 0.040).

### 3.3. Inflammatory Marker Gene Expression Analysis

**Figure 7:**
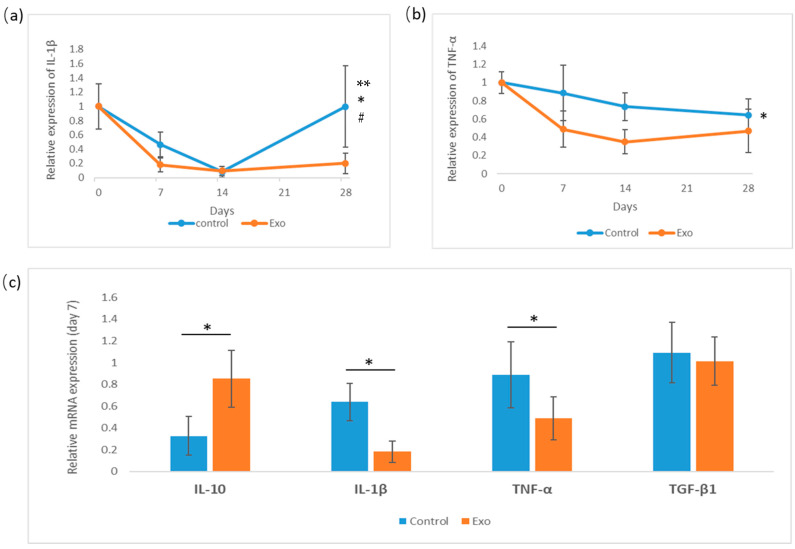
Relative gene expression of cytokines IL-1β, TNF-α analysis for 28 days, and day-7 measurements of IL-10, IL-1β, TNF-α, and TGF-β1. (**a**) Relative gene expression of IL-1β (* group effects, *p* = 0.003, # days effects, *p* = 0.003, ** days × groups interaction effects, *p* = 0.018). (**b**) Relative expression of TNF-α (* group effects, *p* = 0.001). (**c**) Day-7 analysis of cytokines (IL-10 * *p* = 0.025).

The relative mRNA gene expression level of IL-1β, TNF-α, and TGF-β1 in the cornea was analyzed on day 7 of this study. Cytokine IL-1β and TNF-α gene expression levels were monitored for 28 days (Figure 7). IL-1β expression declines sharply in both groups by day 14, with the Exo group showing an earlier and more significant decline. After day 14, the control group’s expression increased and spiked at day 28. The main effects between these groups were significant, F (1, 18) = 11.481, *p* = 0.003, η^2^_p_ = 0.389, with the pairwise comparison showing a significant mean difference of −0.267, SE = 0.097, *p* = 0.011, indicating IL-1β expression in the Exo group was less compared to the control group. The main effects of days were also significant, F (2, 18) = 7.880, *p* = 0.003, η^2^_p_ = 0.467, indicating IL-1β expression changed significantly over time. The group c days interaction was tested as significant, F (2, 18) = 5.103, *p* = 0.018, η^2^_p_ = 0.362, indicating the groups did not have similar trends throughout 28 days of study.

The TNF-α gene expression level declined steadily in both groups between days 7 and 28, with the Exo group being consistently lower. The main effects of the group were tested as significant, F (1, 18) = 14.782, *p* = 0.001, η^2^_p_ = 0.451, with pairwise comparisons showing that the Exo group had less TNF-α gene expression with a significant mean difference, MD = −0.299, SE = 0.081, *p* = 0.001. However, the main effects of day and day × treatment interaction were not significant. This indicates that TNF-α expression does not change significantly over time across all subjects, and both groups share a similar pattern of expression at different time points.

The gene expression of IL-10 and TGF-β1 was analyzed on day 7. An independent *t*-test was conducted to compare IL-10 and TGF-β1 expression between groups on day 7. TGF-β1 expression tested as non-significant. IL-10 expression tested as significant; the Exo group had a significantly higher expression (M = 0.851, SD = 0.260) compared to the control group (M = 0.401, SD = 0.156), t (6) = −2.966, *p* = 0.025.

## 4. Discussion

We successfully established a DED animal model through extra-orbital and infra-orbital lacrimal gland resections. The model exhibited clinical signs and pathological changes that were consistent with severe ocular inflammation. Shinomiya et al. performed the mentioned lacrimal gland resection in mice and observed similar trends of keratoconjunctivitis manifestation [31]. Though both models present a low and minimum tear volume chronically, our model showed a recovery trend of fluorescein staining entering the second month of this study compared to them. We speculated that this was due to species variation, even though by 56 days there was still minimal corneal abrasion in our study. During the onset of aqueous tear deficiency, high levels of inflammatory cytokines (IL-1β, IL-6, TNF-α) contribute to epithelial breakdown [34]. However, adaptive immune responses, such as increased mucin and lipid production, improved lubrication and reduced mechanical trauma [35]. Despite the persistent dryness, epithelial cellular migration and proliferation may adapt and cover the void.

Unlike the clinically occurring DED that involved the lacrimal gland apparatus (which contributes to significant inflammatory cytokines), our study concentrated on observing the hyperosmolarity and desiccation pathogenesis. Hyperosmolarity stresses the corneal epithelial cells, initiates the innate immune response, then triggers the adaptive response that, when prolonged, becomes maladaptive and drives disease progression [36,37,38]. Corneal epithelial cells detect hyperosmolarity and dehydration via surface receptors and ion channels. A severe lack of aqueous tears overwhelmed the osmoprotective pathways on the epithelial cell surface, leading to cell shrinkage, mitochondrial dysfunction, and oxidative stress. These changes compromise cellular metabolism and corneal epithelium integrity [37,38].

Human mesenchymal stem cell-derived exosomes (hMSC-exo) exhibit significant therapeutic potential across species, including in rat models, primarily due to their acellular nature, conserved molecular cargo, and low immunogenicity [39,40]. These nanosized vesicles carry functional RNAs (notably miRNAs), proteins, and lipids that modulate recipient cell behavior, enabling immunomodulation, tissue regeneration, and anti-inflammatory effects in xenogeneic hosts [39,41]. Unlike whole-cell transplants, which often provoke antibody-mediated rejection, hMSC-exosomes lack major histocompatibility complex (MHC) molecules, resulting in the negligible induction of anti-human IgM or IgG antibodies in immunocompetent rats, even after repeated administration [42,43].

UCMSC-exos presented good immunoregulatory effects in our DED model. MSC-Exos exert potent anti-inflammatory effects by reprogramming immune cell activity on the ocular surface. In benzalkonium chloride (BAC)-induced and hypertonic solution-induced DED models, exosomes derived from MSCs significantly reduced the corneal levels of IL-1β and TNF-α by inhibiting the NLRP3 inflammasome pathway. In our case, both IL-1β and TNF-α were suppressed throughout this study. Due to insufficient resources, IL-10 gene expression was only monitored on day 7, though the application of UCMSC-exos for 1 week is sufficient to significantly increase IL-10 expression in the cornea. In other studies, MSC-exos are proven to promote the polarization of M1 macrophages toward the M2 phenotype, which was seen as the increased secretion of IL-10 and arginase-1 (Arg1) [44,45]. Roy et al. showed that higher concentrations of IL-10 in tears are associated with reduced corneal staining, suggesting a protective role against epithelial damage [46]. In our experiment, the therapeutic effects of IL-10 were seen in the significant reduction in the fluorescein staining area and the low apoptotic cell counts. In addition to cytokine modulation, MSC-exos exert a potent inhibitory effect on the proliferation of stimulated T cells (both CD4^+^ and CD8^+^ T cells) [47]. They induce cell cycle arrest by increasing the proportion of cells in the sub-G1 and G0/G1 phases while reducing the S-phase population, mediated through the upregulation of p27^kip1^ and downregulation of Cdk2 proteins [47,48]. Furthermore, MSC-exos enhance the differentiation and immunosuppressive capacity of regulatory T cells (Tregs), partly via the exosomal miRNA-181a-mediated suppression of c-Fos expression [49]. These mechanisms reinforce the broad immunoregulatory role of MSC-exos on the ocular surface, contributing to inflammation resolution and epithelial healing.

The cross-species efficacy of hMSC-exos is supported by the evolutionary conservation of key signaling molecules and pathways [41]. For example, exosomal miRNAs, such as miR-21 and miR-146a, regulate inflammatory pathways (e.g., NF-κB, MAPK) in rat models of traumatic brain injury and osteoarthritis, demonstrating functional homology despite minor sequence mismatches [41]. Moreover, hMSC-exos promote osteogenesis, angiogenesis, and neurogenesis in rats by activating conserved pathways, like BMP/Smad and Wnt/β-catenin, facilitating tissue repair and regeneration [41,50,51]. MSC-exos protect corneal epithelial cells from hyperosmolarity- and inflammation-induced apoptosis. In BAC-treated mice, mADSC-Exos decreased TUNEL-positive cells in the corneal epithelium by 40% compared to the controls, correlating with reduced caspase-3 activation and BAX/BCL-2 ratio [52,53]. In our study, UCMSC-exos effectively suppressed apoptotic cells throughout the 56 days of study. Another study demonstrated that miR-223-3p-enriched exosomes rescued corneal epithelial cells from oxidative stress-induced apoptosis by targeting Fbxw7, a protein implicated in cell cycle arrest [54]. These findings underscore the role of exosomal cargo in preserving epithelial viability under pathological conditions.

Upon encountering severe stress and an inflammatory response, epithelial cells initiate a migration toward the injury sites to restore barrier integrity. This process is followed by cell proliferation, then the reformation of cell–cell junctions and the re-establishment of adhesion to the epithelial basement membrane [55,56,57]. Compared to normal epithelial thickness, the UCMSC-exos in our case modulated epithelial thickening in the treatment group, maintaining it at a minimum level within 2 weeks. MSC-exos restore epithelial uniformity by accelerating wound closure and reducing oxidative damage. For example, ascorbic acid-coupled exosomes (mExo@AA) enhanced corneal epithelial regeneration in murine DED models, achieving a 2.1-fold increase in the healing rate compared to untreated eyes [58]. We also strongly believe that the prominent difference in epithelial thickness was attributed to the suppression of inflammation, hence inflicting less damage to the epithelium.

Although there was no difference in stromal thickness between our treatment and control groups, MSC-exos indirectly preserved stromal homeostasis by mitigating inflammation and epithelial–stromal interactions, reducing IL-1β and TNF-α levels, minimizing matrix metalloproteinase (MMP) activation, and preventing collagen degradation and stromal thinning [52,53].

UCMSC-exos play an important role in anti-fibrosis. In our study, the α-SMA immunostained myofibroblasts in the Exo group increased by day 14, but reduced to a minimum after a month, while the control group had a delayed but intense activation by day 28 and was persistently high through the remainder of this study. MSC-Exos can inhibit the activation of SMAD proteins, which are downstream effectors of TGF-β1 signaling. This inhibition reduces the expression of fibrotic markers, such as α-smooth muscle actin (α-SMA) and collagen type I, thereby mitigating fibrotic tissue remodeling [59,60]. Also, microRNAs in MSC-exos were proven to regulate fibrogenesis. For example, miR-133a has been shown to suppress TGF-β signaling by targeting key fibrotic genes, thus reducing fibrosis progression [61]. Similarly, miR-486-5p MSC-Exos can promote autophagy and reduce fibrosis by modulating key autophagy-related proteins [62].

Importantly, another prominent effect of UCMSC-exos exerted in our animal model is their anti-angiogenic properties. The total length of blood vessels across this study was low compared to the control group. Effects were seen distinctively during the first month of study. Studies have shown that MSC-exos contain anti-angiogenic factors or regulatory molecules that can modulate genes involved in blood vessel formation. For instance, MSC-derived products have been found to contain high concentrations of vascular endothelial growth factor receptor 1 (VEGFR1) [63]. VEGFR1 functions as a decoy receptor for vascular endothelial growth factor (VEGF), sequestering this pro-angiogenic factor and preventing its interaction with VEGFR2, which primarily mediates the pro-angiogenic signaling cascade. Through this mechanism, MSC-Exos may effectively suppress VEGF-driven neovascularization in the cornea. The immunomodulatory properties of MSC-Exos also contribute significantly to their anti-angiogenic effects. By modulating the phenotype and function of infiltrating immune cells, particularly macrophages, MSC-Exos can reshape the inflammatory microenvironment that typically promotes pathological angiogenesis [30]. MSC-Exos have demonstrated the ability to shift macrophages from a pro-inflammatory M1 phenotype toward an anti-inflammatory M2 phenotype, which drives angiogenesis by secreting vascular endothelial growth factor (VEGF), matrix metalloproteinases (MMPs), and transforming growth factor-β (TGF-β), which collectively enhance endothelial cell migration and tube formation [64]. Exosomal CD73/NT5E activity has been identified as a critical mechanism for M2 polarization. It converts AMP to adenosine, activating AKT/ERK-dependent signaling pathways, and promoting M2 polarization [65].

Moreover, MSC-exos also disrupts neutrophil recruitment through neutrophils chemotaxis suppression, reducing neutrophil extracellular trap (NET) formation, as well as neutrophil apoptosis modulation. A study by Arabpour et al. showed that MSC-exos downregulate chemokines and cytokines, such as IL-8 and TNF-α, which are critical for recruiting neutrophils [66]. miR-125a-3p in MSC-exos has been shown to inhibit NET formation, thereby limiting excessive inflammation and tissue injury [67]. Taghavi-Farahabadi, M. and Mahmoudi, M. discovered that MSC-exos can enhance apoptosis through calpain inhibition, reducing prolonged neutrophil activity [68]. An in vitro study showed (miR-199 containing) MSC-exos mitigate neutrophil extracellular trap (NET) formation induced by inflammatory stimuli, like LPS (ex vivo) and PMA (in vitro), decreasing NET formation in mouse neutrophils [67,69]. In our model, though there was a significant overall suppression of neutrophils throughout this study, we observed an elevation of the neutrophil count during the first 2 weeks in the treatment group. We speculated that, due to the severity of our model, the UCMSC-exos may be dose-dependent.

There are some limitations in this study, where more thorough molecular studies, particularly the modulators of the extracellular matrix and mucosal barrier integrity, can be conducted. Matrix metalloproteinases (MMPs), especially MMP-9 and MMP-2 in our case, play a crucial role in corneal epithelial repair and inflammation and were not assessed, leaving a gap in our understanding of the extent of tissue remodeling and degradation. Furthermore, this study lacks an evaluation of mucin markers (such as MUC1, MUC4), which are essential for maintaining tear film stability and epithelial homeostasis. Given the critical role of mucins in ocular-surface protection, their inclusion would provide valuable insights into the therapeutic effects of the intervention on mucosal health. In order to further understand the mechanisms behind epithelial healing and surface integrity, the future research should include MMP profiling and mucin-marker characterization.

## 5. Conclusions

This study of UCMSC-exos application on a dry eye rat model demonstrates significant therapeutic potential in protecting corneal epithelial cells and mitigating pathological changes associated with dry eye disease. Our findings show that treatment with UCMSC-exos effectively reduces inflammatory marker expression, attenuates epithelial apoptosis, and suppresses myofibroblast activation and differentiation, thereby limiting fibrosis and promoting tissue homeostasis. These findings highlight the regenerative and immunomodulatory capabilities of MSC-exosomes in preserving ocular-surface integrity in DED. By reducing inflammatory markers and innate immune cell activation, MSC-Exos alleviate the chronic inflammation that exacerbates DED symptoms. The decrease in apoptotic cells indicates that MSC-Exos promote cell survival, which is essential for maintaining tissue health. Furthermore, the reduction in myofibroblast formation suggests that MSC-Exos may prevent fibrotic changes in the cornea that can lead to scarring and impede vision. Future studies should focus on elucidating the precise molecular mechanisms underlying these effects and optimizing therapeutic strategies for clinical translation in dry eye disease management.

## Data Availability

The data presented in this study are available on request from the corresponding authors.

## Supplementary Materials

The following supporting information can be downloaded at: https://www.mdpi.com/article/10.3390/biomedicines13051174/s1, Figure S1. Corneal neovascularization and fluorescein staining of the control, Exo, and control + PBS group. (a) Photography images of corneal vascularization progression of the three groups from day of lacrimal gland excision (day -2) to day 14. The blood vessels length of the control and control + PBS group showed a similar trend throughout 14 days. (b) Corneal neovascularization length (mm) changes along 14 days of study. Both the control and control + PBS group were tested significantly different from the Exo group. (* *p* = 0.003, # *p* = 0.032). (c) Photography images of corneal fluorescein staining of the three groups from day of lacrimal gland resection to day 14. Both the control and control + PBS groups showed similar trend throughout 14 days. (d) Corneal fluorescein staining area (mm^2^) throughout 14 days of study. Both the control and control + PBS group were tested dignificantly from the Exo group. (* *p* = 0.043, # *p* = 0.023).; Figure S2: Corneal epithelium and stromal thickness examination and analysis throughout 14 days comparing the control, Exo and control+PBS groups. (a) OCT images of the cornea. Both the control and the control+PBS groups had more thickening of epithelium day 7 and 14. (b) Epithelium thickness of the three groups. * different from Exo (*p* = 0.003), # different from norm (*p* = 0.003), ** different from Exo (*p* = 0.003), ## different from norm (p = 0.005). (c) Stromal Thickness of the three groups. *different from norm (*p* < 0.001). Figure S3. (a) Histopathology H&E staining and (b) neutrophil cell count analysis. Figure S4: Corneal epithelial and stromal thickness measurement via OCT images. A representative image of a normal cornea with epithelium and stromal intact. Measurements were taken at the central point of the cornea, and 1000 µm medial and lateral side from the central point. Measurements of the thickness was calculated as mean of these 3 measurement points.; List S1: The primer sequences used in this study.

## Abbreviations

The following abbreviations are used in this manuscript:

MSC	Mesenchymal stem cell
MSC-exos	Mesenchymal stem cell-derived exosomes
UCMSC-exos	Umbilical cord mesenchymal stem cell-derived exosomes
hMSC-exos	Human mesenchymal stem cell-derived exosomes
DED	Dry eye disease
NK cells	Natural killer cells
IFN-γ	Interferon-gamma
TNF-α	Tumor necrosis factor-alpha
