# Supplementary material for: Therapeutic Potential of Umbilical Cord MSC-Derived Exosomes in a Severe Dry Eye Rat Model: Enhancing Corneal Protection and Modulating Inflammation
Source: Biomedicines. 2025 May 11;13(5):1174. doi: 10.3390/biomedicines13051174 (PMC12108626; doi:10.3390/biomedicines13051174)
Supplement: Supplementary file 1 [file biomedicines-13-01174-s001.zip › biomedicines-3575443-supplementary.pdf]

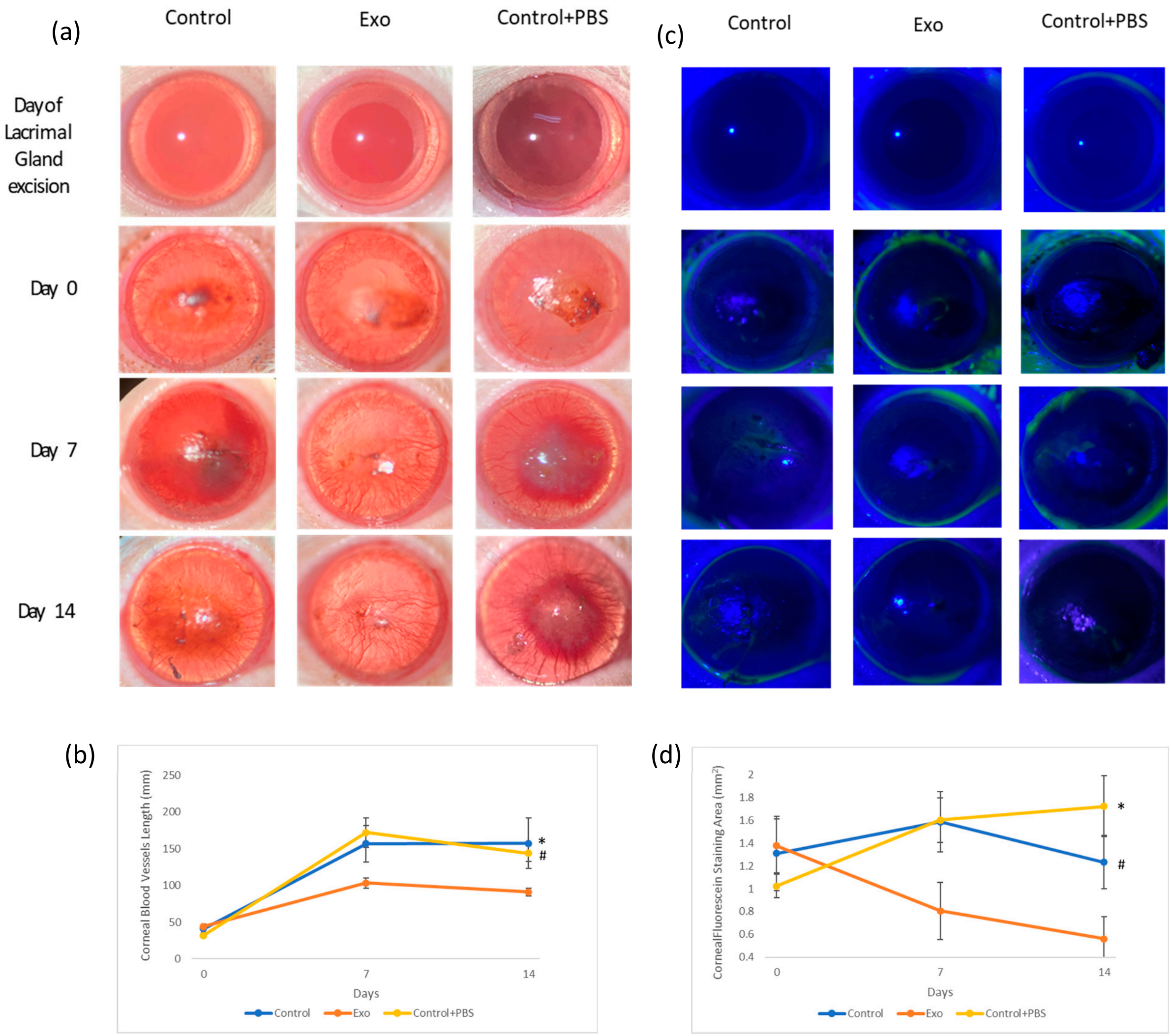

Figure S1. Corneal neovascularization and fluorescein staining of the control, Exo, and control + PBS group. (a) Photography images of corneal vascularization progression of the three groups from day of lacrimal gland excision (day -2) to day 14. The blood vessels length of the control and control + PBS group showed a similar trend throughout 14 days. (b) Corneal neovascularization length (mm) changes along 14 days of study. Both the control and control + PBS group were tested significantly different from the Exo group. (* p = 0.003, # p = 0.032). (c) Photography images of corneal fluorescein staining of the three groups from day of lacrimal gland resection to day 14. Both the control and control + PBS groups showed similar trend throughout 14 days. (d) Corneal fluorescein staining area (mm^2) throughout 14 days of study. Both the control and control + PBS group were tested and found to be insignificantly different from the Exo group. (* p = 0.043, # p = 0.023).

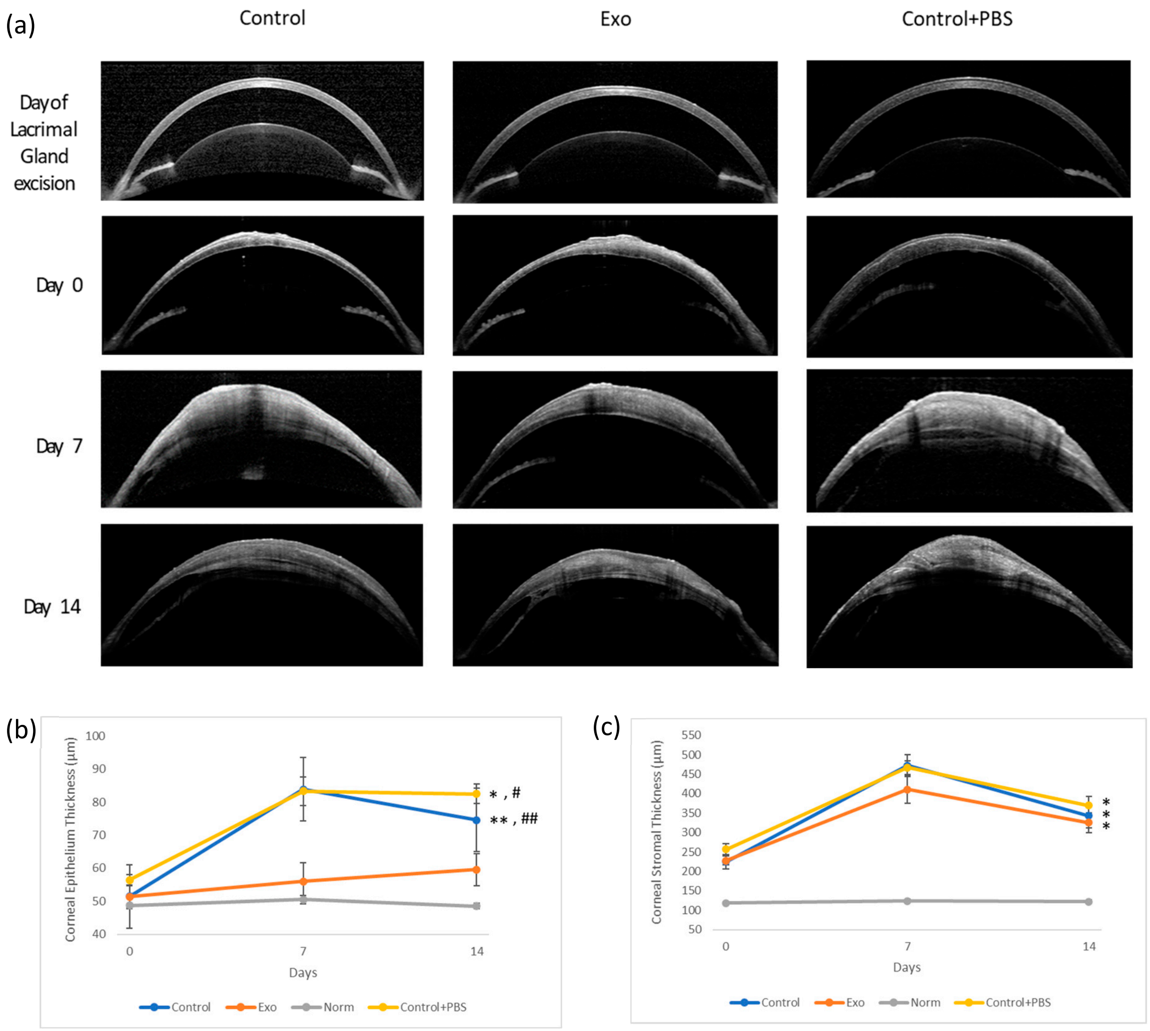

Figure S2. Corneal epithelium and stromal thickness examination and analysis throughout 14 days comparing the control, Exo and the control+PBS groups. (a) OCT images of the cornea. Both the control and the control + PBS groups had more thickening of the epithelium days 7 and 14. (b) Epithelium thickness of the three groups. * different from Exo ($p = 0.003$), # different from norm ($p = 0.003$), ** different from Exo ($p = 0.003$), ## different from norm ($p = 0.005$). (c) Stromal Thickness of the three groups. *different from norm ($p < 0.001$).

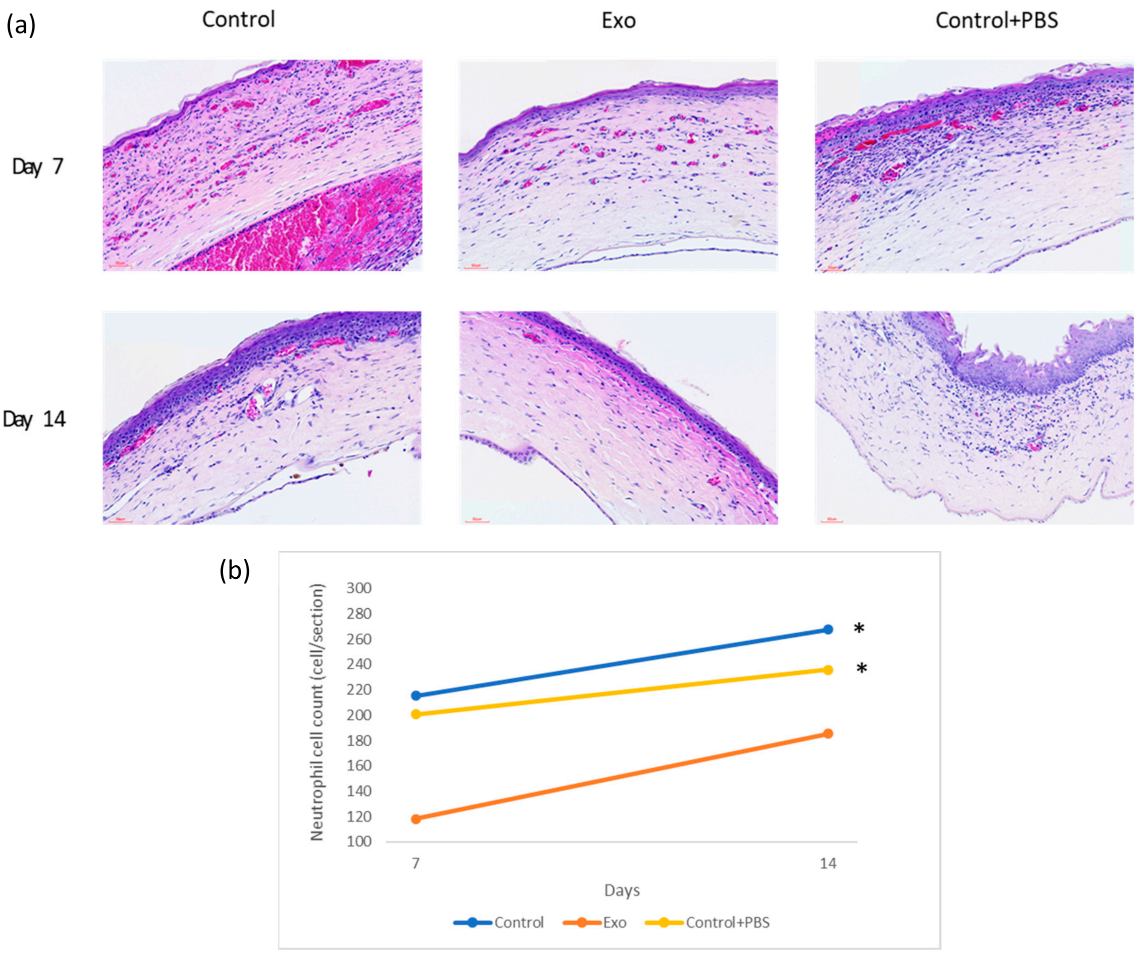

Figure S3. (a) Histopathology H&E staining and (b) neutrophil cell count analysis. Neutrophils infiltrated mainly at the anterior half of the stroma, predominantly immediately below the epithelial basement membrane. * different from Exo group ($p < 0.001$)

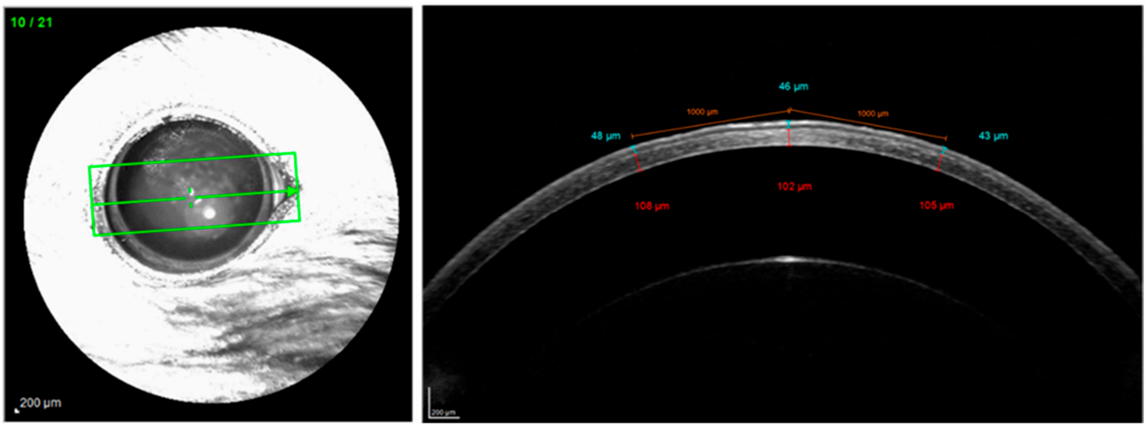

Figure S4. Corneal epithelial and stromal thickness measurement via OCT images. A representative image of a normal cornea with epithelium and stromal intact. Measurements were taken at the central point of the cornea, and 1000 μm medial and lateral sides from the central point. Measurements of the thickness were calculated as the mean of these 3 measurement points.

Rat TGF beta 1 qPCR Primer Pair (SinoBiological, Cat: RP300111)

Rat TNF-alpha qPCR Primer Pair (SinoBiological, Cat: RP300044)

Rat IL-1 beta qPCR Primer Pair (SinoBiological, Cat: RP300022)

Rat IL-10 qPCR Primer Pair (SinoBiological, Cat: RP300078)

mRNA Gene	Forward (5 ′-3 ′)	Reverse (3 ′-5 ′)
GAPDH	GCAAGGATACTGAGAGCAAGAG	GGATGGAATTGTGAGGGAGATG

List S1. The primer sequences used in this study.
